# The physical activity and nutrition-related corporate social responsibility initiatives of food and beverage companies in Canada and implications for public health

**DOI:** 10.1186/s12889-020-09030-8

**Published:** 2020-06-09

**Authors:** Monique Potvin Kent, Elise Pauzé, Kevin Guo, Arianne Kent, Royce Jean-Louis

**Affiliations:** 1 School of Epidemiology and Public Health, Faculty of Medicine, University of Ottawa, 600 Peter Morand, room 301J, Ottawa, Ontario K1G5Z3 Canada; 2 Faculty of Medicine, University of Ottawa, 451 Smyth Rd, Ottawa, Ontario K1H 8M5 Canada; 3 Department of Sociology, Faculty of Arts, McGill University, 855 Sherbrooke Street West, Montreal, Quebec, H3A 2T7 Canada; 4 Interdisciplinary School of Health Sciences, Faculty of Health Sciences, University of Ottawa, 25 University Private, Ottawa, Ontario K1N 7K4 Canada

**Keywords:** Food marketing, Children, Political activity, Self-regulation, Conflicts of interest

## Abstract

**Background:**

As diet-related diseases have increased over the past decades, large food companies have come under scrutiny for contributing to this public health crisis. In response, the food industry has implemented Corporate Social Responsibility (CSR) initiatives related to nutrition and physical activity to emphasize their concern for consumers. This study sought to describe the nature and targeted demographic of physical activity and nutrition-related CSR initiatives of large food companies in Canada and to compare companies who participate in the Canadian Children’s Food and Beverage Advertising Initiative (CAI), a self-regulatory initiative aimed at reducing unhealthy food advertising to children, with non-participating companies.

**Methods:**

A cross-sectional study was conducted in 2016. Thirty-nine large food companies, including 18 participating in the CAI, were included in the study. The webpages, Facebook pages and corporate reports of these companies were surveyed to identify CSR initiatives related to nutrition and physical activity. Initiatives were then classified by type (as either philanthropic, education-oriented, research-oriented or other) and by targeted demographic (i.e. targeted at children under 18 years or the general population). Differences between CAI and non-CAI companies were tested using chi-square and Mann-Whitney U tests.

**Results:**

Overall, 63 CSR initiatives were identified; 39 were nutrition-related while 24 were physical activity-related. Most (70%) initiatives were considered philanthropic activities, followed by education-oriented (20%), research-oriented (8%) and other (2%). Almost half (47%; *n* = 29) of initiatives targeted children. Examples of child-targeted initiatives included support of school milk programs (n = 2), the sponsorship of children’s sports programs (*n* = 2) and the development of educational resources for teachers (*n* = 1). There were no statistically significant differences in the number of CSR initiatives per company (CAI: Mdn = 1, IQR = 3; non-CAI: Mdn = 0, IQR = 2; *p* = .183) or the proportion of child-targeted initiatives (CAI: 42%; non-CAI: 54%; *p* = .343) between CAI and non-CAI companies.

**Conclusion:**

Food companies, including many that largely sell and market unhealthy products, are heavily involved in physical activity and nutrition-related initiatives in Canada, many of which are targeted to children. Government policies aimed at protecting children from unhealthy food marketing should consider including CSR initiatives that expose children to food company branding.

## Background

Alarmingly, rates of obesity and nutrition-related noncommunicable diseases (NCDs) have been increasing globally in the past few decades [1–3]. In Canada, the percentage of the childhood population aged 13 years and younger with obesity has tripled between 1981 and 2011 [4]. Although childhood obesity rates have plateaued in high-income countries in recent years, they continue to increase dramatically in low and middle-income countries [1]. These trends are concerning as obesity, in addition to being a disease, is also a major risk factor for other serious illnesses such as diabetes, liver and cardiovascular disease, and certain cancers [5, 6]. In addition to the social inequities which contribute to poor diet and obesity [7, 8], the predominantly unhealthy nature of the food supply is likely a major contributor to this public health crisis [9, 10]. As a result, large food and beverage companies have been criticized for their role in the development of these diseases [6, 11].

In response to mounting scrutiny, the food industry has adopted self-regulatory measures and initiatives to emphasize their concerns about the health and well-being of consumers [12]. For many companies, these activities fall under their Corporate Social Responsibility (CSR) which they define as their “economic, legal, ethical, and philanthropic responsibilities to society and their shareholders” [13]. Many CSR activities put forth by large food and beverage companies are related to physical activity and nutrition. These include providing nutritional information for consumer education, selling healthier food alternatives, partnering with non-profit organizations and charities, sponsoring community activities and sports programs, and funding research in the fields of nutrition and physical activity [12].

Numerous issues have been raised regarding the effectiveness and genuine intentions of these programs with some suggesting that these activities are in fact marketing or public relation tactics employed by companies to portray themselves in a positive light [12, 14–18]. For instance, the unhealthy nature of many items marketed by these companies runs contradictory to the healthy living ideals of many of their CSR initiatives. Research has shown that CSR programs can create a “health halo” and associate companies with positive health attributes [15]. Others have also drawn parallels between the food industry’s self-regulatory measures and those employed by the tobacco industry decades ago to deflect criticism and delay government intervention [12, 19, 20]. One such initiative includes the voluntary restriction of unhealthy food and beverage advertising to children. Since the late 2000’s, food and beverage companies in the United States, Australia, Norway, and Canada, among others, have developed self-regulatory (industry-led) or co-regulatory arrangements (i.e. self-regulation developed with or requested by government) to limit unhealthy food and beverage marketing to children [21, 22]. In Canada, for example, the Children’s Food & Beverage Advertising Initiative (CAI) involves 18 large food and beverage companies who have voluntarily pledged to not advertise unhealthy foods to children younger than 12 years [23].

While a few studies have characterized food industry CSR initiatives, particularly in Australia [12, 24], no such research has been done in Canada. The purpose of the current study was to examine the nature and targeted demographic of physical activity and nutrition-related CSR initiatives of large food and beverage companies in Canada and to compare the initiatives of CAI companies and those of non-CAI companies who advertise to children. It was hypothesized that companies participating in the CAI would be less likely to be involved in CSR activities that are child-targeted given their commitment to reduce marketing to children.

## Methods

### Study sample and data collection

This study examined the CSR initiatives of 39 companies including the 18 food and beverage companies participating in the CAI in 2016 and 21 non-participating companies [25]. The latter companies were selected from May 2013 Neilsen Media Research data, based on their rank as the top food and beverage advertisers on 4 selected children and youth television stations broadcasted in Toronto, the most populous city in Canada. To identify CSR initiatives, research assistants consulted the Canadian website of each company (A.K.), their associated Canadian brand websites (A.K.), their Canadian Facebook pages going back 1 year (A.K.), if available, and their Canadian annual corporate reports (K.G.) and recorded their description. Data from Facebook pages and websites were collected from June to August 2016 while corporate CSR reports from 2016 were reviewed in April 2018.

When identifying initiatives, the following information was collected from the websites and inputted into a spreadsheet, if available: the name of the food and beverage/fast food company, the name of the initiative, the date of initiative announcement, the product highlighted or advertised through the initiative (i.e. the specific brand involved), the collaborators (e.g. community associations, school programs, non-profit organizations, government, universities etc.), and a brief summary of the initiative (found on the company website, Facebook page or on the sponsored activity’s website).

### Content analysis

Identified CSR initiatives were categorized as either physical activity or nutrition-related. Initiatives related to physical activity included those related to sports or physical activity (for example, sponsorship of sporting events) while nutrition-related initiatives included any initiative related to nutrition or healthy eating (for example, funding nutrition research, donations to breakfast programs or the dissemination of nutrition information). If a CSR initiative was related to both nutrition and physical activity, it was coded based on the component that was sponsored by the company or, if the latter did not apply, based on the predominant nature of the initiative. If the physical activity-based initiatives mentioned the use of professional athletes as ambassadors, this was also noted.

An initiative was coded as child-targeted if “children”, “teen”, or “youth” were explicitly mentioned in the initiative description, if the targeted age group included children aged 17 and under, if the sponsored activity implied that children were targeted (e.g. sponsorship of initiatives in schools, during spring break or involved the development of branded school materials) or if the activity supported children’s charities (e.g. UNICEF). The remaining initiatives were considered targeted at the general population.

CSR initiatives were also coded into categories describing the type of activities supported or conducted by the food and beverage company. They were categorised as “education-oriented” if they aimed to disseminate information about nutrition or physical activity to consumers or health professionals (e.g. blogs/recipes, websites about nutrition, ad campaigns), as “research-oriented” if they involved funding research or as “charity/philanthropy” if the initiative supported or sponsored programs, events, charities, organizations, or fundraisers related to nutrition or physical activity (e.g. charitable donations to food banks, sponsorship of sports teams and school programs, etc.). Initiatives that did not fall into these categories were categorized as “other”.

The CSR initiatives were coded by two-trained research assistants (E.P. and K.G.). The inter-rater reliability for the nature of the initiative (nutrition or physical activity), the target population (children or general), and the type of initiative (education, research, charity/philanthropy or other) were 97, 89, and 87%, respectively. These were calculated using the following formula: 1 - (*n* disagreements/63 initiatives). Disagreements were reviewed by the lead author (MPK) and one of the primary coders (E.P.) and the correct coding was determined through discussion. Statistical analyses were conducted using SPSS version 25.

Identified initiatives were described using frequencies. The average and median number of CSR initiatives per company was determined and the characteristics of these initiatives were described using frequencies. A qualitative description of CSR initiatives is also provided by type of initiative. The difference in the number of CSR initiatives per company by CAI participation was examined using a Mann-Whitney U test while differences in the proportion of child-targeted initiatives and nutrition versus physical activity related initiatives were examined using chi-square tests. A *p*-value lower than 0.05 was considered statistically significant. Examples of products promoted through CSR initiatives are provided by CAI and non-CAI companies.

## Results

A total of 157 websites, 131 Facebook pages, and 5 reports were identified and analyzed as shown in Table 1. The list of consulted websites, Facebook pages and Canadian annual reports are listed in Supplementary Table 1.

**Table 1:** Total number of websites, Facebook pages and reports analyzed for CAI and non-CAI food and beverage companies

	**Food and Beverage Company**	**Websites Analyzed*n*(%)**	**Facebook Pages Analyzed*n*(%)**	**Corporate Reports Analyzed*n*(%)**	**Total** ***n*(%)**
CAI Companies	Campbell Company of Canada	1 (1.2)	2 (2.3)	0 (0)	3 (1.7)
	Coca-Cola Canada Ltd.	1 (1.2)	1 (1.2)	0 (0)	2 (1.2)
	Danone Canada Inc.	3 (3.5)	4 (4.7)	0 (0)	7 (4.1)
	Ferrero Canada Ltd.	4 (4.7)	4 (4.7)	0 (0)	8 (4.6)
	General Mills Canada Corporation	3 (3.5)	2 (2.3)	0 (0)	5 (2.9)
	Hershey Canada Inc.	2 (2.3)	4 (4.7)	0 (0)	6 (3.5)
	Kellogg’s Canada Inc.	7 (8.2)	5 (5.9)	0 (0)	12 (7.0)
	Kraft Canada Inc.	4 (4.7)	5 (5.9)	0 (0)	9 (5.2)
	Maple Leaf Foods	6 (7.0)	6 (7.0)	1 (50.0)	13 (7.6)
	Mars Canada	1 (1.2)	2 (2.3)	0 (0)	3 (1.7)
	McDonald’s Restaurants of Canada Limited	1 (1.2)	1 (1.2)	0 (0)	2 (1.2)
	Mondelēz Canada	2 (2.4)	2 (3.4)	0 (0)	4 (2.3)
	Nestlé	8 (9.4)	14 (16.5)	0 (0)	22 (12.8)
	Parmalat Canada	10 (11.8)	7 (8.2)	0 (0)	17 (9.9)
	Pepsico Canada	17 (20.0)	15 (17.6)	0 (0)	32 (18.6)
	Post Foods Canada Corporation	1 (1.2)	1 (1.2)	0 (0)	2 (1.2)
	Unilever Food Division	5 (5.9)	5 (5.9)	0 (0)	10 (5.8)
	Weston Foods Canada	9 (10.6)	5 (5.9)	1 (50.0)	15 (8.7)
	**Total (100)**	**85 (100)**	**85 (100)**	**2 (100)**	**172 (100)**
Non-CAI Companies	Agropur Co-Op	15 (20.8)	6 (13.0)	1 (33.3)	22 (18.1)
	Arla Foods Inc.	3 (4.2)	3 (6.5)	0 (0)	6 (5.0)
	A&W Food Services of Canada Inc.	1 (1.4)	1 (2.2)	0 (0)	2 (1.7)
	Burger King Corp.	1 (1.4)	1 (2.2)	0 (0)	2 (1.7)
	Cara Operations LTD.	11 (15.3)	9 (19.5)	1 (33.3)	21 (17.3)
	Con-Agra Foods Canada Inc.	9 (12.5)	5 (10.9)	0 (0)	14 (11.6)
	Dairy Farmers of Canada	6 (8.3)	1 (2.2)	1 (33.3)	8 (6.6)
	Dairy Queen Restaurant	1 (1.4)	1 (2.2)	0 (0)	2 (1.7)
	Dare Foods	1 (1.4)	5 (10.9)	0 (0)	6 (5.0)
	Darden Restaurants Inc.	1 (1.4)	0 (0)	0 (0)	1 (0.8)
	Canada Dry Mott’s Inc. (Dr. Pepper Snapple Group)	7 (9.7)	5 (10.9)	0 (0)	12 (9.9)
	Fromageries Bel Sa	4 (5.5)	1 (2.2)	0 (0)	5 (4.1)
	Hormel Foods	1 (1.4)	1 (2.2)	0 (0)	2 (1.7)
	Mccain Foods Canada 1	1 (1.4)	1 (2.2)	0 (0)	2 (1.7)
	Quizno’s Corp.	1 (1.4)	1 (2.2)	0 (0)	2 (1.7)
	RedBull LTD	1 (1.4)	0 (0)	0 (0)	1 (0.8)
	Storck International	1 (1.4)	0 (0)	0 (0)	1 (0.8)
	Subway Canada	1 (1.4)	1 (2.2)	0 (0)	2 (1.7)
	Tim Horton’s Canada	1 (1.4)	1 (2.2)	0 (0)	2 (1.7)
	Wendy’s company (Triarc)	1 (1.4)	1 (2.2)	0 (0)	2 (1.7)
	Yum! Brands Inc	4 (5.5)	2 (4.3)	0 (0)	6 (5.0)
	**Total (100)**	**72 (100)**	**46 (100)**	**3 (100)**	**121 (100)**

### Frequency and nature of identified CSR initiatives

Overall, it was identified that the 39 companies participated in 63 CSR initiatives, of which 29 (47%) were child-targeted. Twenty-four (38%) CSR initiatives were physical activity-related while 39 (62%) were nutrition-related. Most identified CSR initiatives were considered charitable and philanthropic activities (*n* = 44; 70%), followed by education-oriented (*n* = 13; 20%), research-related (*n* = 5; 8%) and other (*n* = 1; 2%) (Table 2).

**Table 2:** Characteristics of identified nutrition and physical activity-related corporate social responsibility (CSR) initiatives, overall and by participation in the Canadian Children’s Food and Beverage Advertising Initiative (CAI)

	**CAI companies ** **(*N*** **= 36 initiatives)**	**Non-CAI companies ** **(*N*** **= 27 initiatives)**	**All companies ** **(*N*** **= 63 initiatives)**
**Number of identified CSR initiatives**^**†**^			
Mean (SD)	2.0 (2.4)	1.3 (2.4)	1.6 (2.4)
Median (IQR)	1 (3)	0 (2)	1 (2)
Min-Max	0–7	0–9	0–9
	***n*(%)**	***n*(%)**	***n*(%)**
**Type of CSR initiative**			
Charitable activities / Philanthropy	22 (61)	22 (81)	44 (70)
Education-oriented	9 (25)	4 (15)	13 (20)
Research-oriented	4 (11)	1 (4)	5 (8)
Other	1 (3)	0 (0)	1 (2)
**Nutrition or Physical activity CSR**^**‡**^			
Nutrition-related	26 (72)	13 (48)	39 (62)
Physical activity related	10 (28)	14 (52)	24 (38)
**Targeting of CSR initiatives**^**§**^			
Child-targeted	15 (42)	14 (54)	29 (47)
General-targeted	21 (58)	12 (46)	33 (53)

^**†**^*SD* Standard deviation, *IQR* Interquartile range. The number of CSR initiatives per company by CAI participation was not statistically significant (U = 237; z = 1.434; p = .183)

^**‡**^ The difference in proportion by CAI participation was not statistically significant (X^2^ = 3.792; df = 1; *p* = .052)

^**§**^ The difference in proportion by CAI participation was not statistically significant (X^2^ = .900.; df = 1; *p* = .343)

### Description of identified CSR initiatives by type of activity

Identified CSR initiatives are described in Table 3. Charitable and philanthropic CSR initiatives related to nutrition (*n* = 20) involved the support of school milk programs (*n* = 2) and various international, national and provincial organizations (e.g. World Food Program, Food Banks Canada, Breakfast Club of Canada, Foundation OLO; *n* = 16) or local programs (e.g. Brandon’s Food For Thought Breakfast and Snack Program; *n* = 2) addressing short-term food security. Forms of support to these organizations included financial contributions, food donations (e.g. milk, yogurt, breakfast cereal), and an industry-led fundraiser (e.g. Toonies for Tummies organized by the Grocery Foundation). Charitable and philanthropic CSR activities related to sports or physical activity (*n* = 24) included corporate philanthropy programs that provide funding or sports equipment to schools, teams or community organizations (e.g. Canada Dry Mott’s ACTION Nation program; Nestlé’s Good Food, Good Life Community Program) (*n* = 3), the distribution of grants to young female athletes or teams (*n* = 1), and the sponsorship of races, events or fundraisers (*n* = 10), children’s minor sports programs (*n* = 2; e.g. Timbits Minor Sports Program) as well as non-profit organisations promoting physical activity among youth (*n* = 2) (e.g. ParticipAction, Grand Défi Pierre Lavoie), among other initiatives. Two companies (Danone & McDonald’s) reported having professional athletes as ambassadors for one of their CSR initiatives targeting children.

**Table 3:** Number of identified corporate social responsibility initiatives by CAI and Non-CAI food and beverage company

**CAI/ Non-CAI**	**Company**	**CSR Initiatives*n*(%)**	**Description of the Initiative**	**Categorization of the initiative**
CAI Companies	Campbell Company of Canada	1 (2.8)	“Help Hunger Disappear”: “To donate food to food banks across Canada and encourage Canadians to get involved and make donations to their local food banks.”	Philanthropy Nutrition General-targeted
	Coca-Cola Canada Ltd.	2 (5.6)	“Donating Minute Maid 100% fruit juices to Breakfast Club of Club of Canada”	Philanthropy Nutrition Child-targeted
			Funding provided to ParticipAction Teen Challenge “A non-profit organization dedicated to inspiring and supporting healthy and active living in Canada.”	Philanthropy Physical activity Child-targeted
	Danone Canada Inc.	7 (19.4)	“Donating yogurt to Breakfast Club of Canada and Breakfast Club of Quebec”	Philanthropy Nutrition Child-targeted
			“Feel Good Everyday Blog Healthy Eating”: “Providing nutritional advice to Danone customers.”	Education Nutrition General-targeted
			“Nutricia Research”: “A new global research centre to accelerate innovation in early life nutrition and advanced medical nutrition.”	Research Nutrition General-targeted
			“Nutrition Facts Education Campaign”: “… a collaboration between Food & Consumer Products of Canada (FCPC) and Health Canada that will better enable Canadians to understand and use the Nutrition Facts table (NFt), particularly the % Daily Value, on packaged food products.”	Education Nutrition General-targeted
			“The Danone Institute of Canada: Yogurt in Nutrition: Initiative for a Balanced Diet”: “A non-profit foundation created in 1998 and funded by Danone inc. It is administered by an independant board of directors and scientific council, comprised largely of members that are dietitians and researchers.” The institute provides research grants and awards.	Research Nutrition General-targeted
			Danone Nations Cup “A chance for the best boys and girls in the age range 10 to 12 year-olds to showcase their soccer talents in a major international soccer competition. Zinedine Zidane is the international Ambassador of the Danone Nations Cup.”	Philanthropy Physical activity Child-targeted
			Lole White Tour “On Wednesday August 10, join us for the very first Oikos Canada #FacebookLive Yoga session and get ready for LOLË: The White Tour Montreal. This live class given by Juna Yoga founder Nadia Bonenfant will start at 8:15 a.m. (ET). Don’t miss it!”	Philanthropy Physical activity General-targeted
	Kellogg’s Canada Inc.	2 (5.6)	“Breakfasts for Better Days”: Program involving the donation of Kellogg’s cereals to “feed children and families across Canada”.	Philanthropy Nutrition Child-targeted
			“Nutrition at its Best From Kellogg’s”: “For more than 100 years, our best is in providing a nourishing start for families everywhere. Delicious, wholesome beginnings that fill bellies and fuel bodies and minds.”	Education Nutrition General-targeted
	Kraft Canada Inc.	2 (5.6)	“Healthy Living”: “Kraft Canada’s selection of Healthy Living recipes helps you plan a delicious and health-conscious meal for you & your family.”	Education Nutrition General-targeted
			“Hockey Centre ”: Nutrition education and recipes for families with hockey-playing kids.	Education Nutrition General-targeted
	Maple Leaf Foods	5 (13.9)	“Tastebuds”: “Tastebuds is a partnership among community agencies that supports local student nutrition programs for children and youth in Hamilton.”	Philanthropy Nutrition Child-targeted
			“Food for Thought”: “In 2015, Brandon’s Food for Thought Breakfast & Snack Program provided a nutritional breakfast or snack at 19 schools, serving more than 72,000 meals to students.”	Philanthropy Nutrition Child-targeted
			UNICEF Canada: “Maple Leaf has a long-standing relationship with UNICEF Canada, helping the organization to leverage funding from other sources and deliver critical nutrition relief to crisis areas around the world.”	Philanthropy Nutrition Child-targeted
			“Advancing Nutrition and Health” “There is significant commercial and social benefit to advancing the nutrition and health benefits of the Company’s products. Maple Leaf Foods continues to advance the use of simpler, natural ingredients, reducing or eliminating antibiotic use in animal production, and other key initiatives including reducing sodium levels to meet Health Canada guidelines”	Other Nutrition General-targeted
			Maple Leaf Centre for Action on Food Security “The Maple Leaf Centre for Action on Food Security (“the Centre”) collaborates with other organizations and individuals to advance food security. We are seeking to raise the profile of this pressing social issue, advocate for critical policies and invest in programs required to make sustainable improvements.” / “MISSISSAUGA, ON, December 6, 2016 – Maple Leaf Foods today announced a long-term commitment to advance sustainable food security through the launch of the Maple Leaf Centre for Action on Food Security (“the Centre”), a not-for-profit organization. The Company expects to invest over $10 million over the next five years to support the Centre’s activities and will also make product donations exceeding $1.5 million annually. (....) The Centre will share learning from its work and support networks, collaboration and research in the food security sector that builds further understanding of the issues, approaches and enables knowledge transfer.”	Philanthropy Nutrition General-targeted
	McDonald’s Canada	1 (2.8)	atoMC/ Equipe McDo (Qc) “McDonald’s Canada is proud to support minor hockey in communities across the country, by offering young players a unique opportunity to build their sense of teamwork and improve both their on-ice game skills and off-ice life skills.” Minor Hockey, Atom Level (10-11y.o.) & Bantom Level (13–14 y.o. (Qc) / 3 AtoMC Athlete Ambassadors	Philanthropy Physical Activity Child-targeted
	Mondelēz Canada	1 (2.8)	“Nutrition Science Corner”: “We design, develop and share our innovations in nutrition research through the foods and beverages we create.”	Education Nutrition General-targeted
	Nestlé	5 (13.9)	“Good Food, Good Life Community Program”: “Nestlé Canada’s *Good Food, Good Life* Community Program encourages and supports the promotion of health and wellness in the communities we all share.”/ “Our charitable and non-profit support is directed toward programs and organizations that support healthy active living, with specific emphasis on encouraging physical activity in children, youth and their families, all in a non-competitive setting.”	Philanthropy Physical Activity Child-targeted
			Donations to Food Banks Canada	Philanthropy Nutrition General-targeted
			“Nutrition Facts Education Campaign”: “… a collaboration between Health Canada and Food & Consumer Products of Canada (FCPC). The initiative is a broad-based campaign launched in 2010 to help Canadians better understand and use the Nutrition Facts table (NFt), particularly the % Daily Value, on packaged food products to make informed food choices.”	Education Nutrition General-targeted
			“Le Grand Défi Pierre Lavoie”: “… an organization committed to promoting healthy life habits among young people, through physical activity and healthy eating … Established in 2008, Le Grand Défi is the largest health-related activity ever organized in the province of Quebec.”	Philanthropy Physical activity Child-targeted
			“EATracker”: “Nestlé Canada wants to help Canadians make the right food and activity choices. EATracker is a tool produced by the Dietitians of Canada (and developed with a technical grant from Nestlé Canada) to help individuals keep track of their daily food and activity choices, and show how they are doing compared to current recommendations.”	Education Nutrition General-targeted
	Parmalat Canada	2 (5.6)	Donation to Breakfast Club of Canada	Philanthropy Nutrition Child-targeted
			“Toonies for Tummies”: “Toonies for Tummies, brought to you by The Grocery Foundation, helps support over 4500 school nutrition programs in Ontario.”	Philanthropy Nutrition Child-targeted
	Pepsico Canada	7 (19.4)	“Nutrition Facts Education Campaign”: “… a collaboration between Food & Consumer Products of Canada (FCPC) and Health Canada that will better enable Canadians to understand and use the Nutrition Facts table (NFt), particularly the % Daily Value, on packaged food products.”	Education Nutrition General-targeted
			Support of The Canadian Foundation for Dietetic Research “The Canadian Foundation for Dietetic Research (CFDR) is a registered charitable foundation that provides grants for research in dietetics and nutrition. This research supports quality advice, programs, resources and service delivery—based on credible scientific evidence—that ultimately enhances the health of Canadians.”	Research Nutrition General-targeted
			“Gatorade Sports Science Centre”: “Founded in 1985, the Gatorade Sports Science Institute (GSSI) is committed to helping athletes optimize their health and performance through research and education in hydration and nutrition science.”	Research Nutrition General-targeted
			Partnership with the YMCA	Philanthropy Physical Activity General-targeted
			Donation to Food Banks Canada	Philanthropy Nutrition General-targeted
			O-Course “The O Course is an EXTREME obstacle course workout designed by former U.S. Marine Drill Instructor Sgt. Tony A. It will challenge you physically and mentally to help you #KEEPSWEATING this summer!”	Philanthropy Physical activity General-targeted
			GCAMP “Gatorade is hosting #GCAMP on July 26 & 27; a hockey camp that will bring together players all across Canada who have demonstrated what it means to #WinFromWithin.”	Philanthropy Physical activity Child-targeted
	Weston Foods Canada	1 (2.8)	“Weston Cares”: “Weston Cares is a National initiative driven by employees to raise money for children’s charities promoting healthy active living.”	Philanthropy Physical activity Child-targeted
	**Total (100)**	**36 (100)**		
Non-CAI Companies	Agropur Co-Op	9 (33.3)		
			“Club des petits déjeuners du Québec”: “… the Club des petits déjeuners du Québec serves hot breakfasts to students, providing two essential ingredients for our youth to start their school day: a nutritious breakfast and a nourishing environment.”	Philanthropy Nutrition Child-targeted
			Donations to The Breakfast Clubs of Canada	Philanthropy Nutrition Child-targeted
			Support of The Foundation OLO “The Foundation OLO helps children get a good start in life and come into this world in good health through appropriate action and nutritional support” This program provides food vouchers and nutritional supplements to pregnant women.	Philanthropy Nutrition General-targeted
			“Milk for Kids”: “… providing fresh milk to families in need through local food banks. Begun in Victoria in 1999, the Milk for Kids program now provides more than 70,000 litres of milk each year to families throughout Vancouver Island.”	Philanthropy Nutrition Child-targeted
			Active Lives (no description available)	Philanthropy Physical activity Unknown target group
			Times Colonist 10 K Run	Philanthropy Physical activity General-targeted
			Supercities Walk for MS	Philanthropy Physical activity General-targeted
			CIBC Run for the Cure	Philanthropy Physical activity General-targeted
			Victoria’s Dragon Boast Festival	Philanthropy Physical activity General-targeted
	Canada Dry Mott’s Inc. (Dr. Pepper Snapple Group)	2 (7.4)	“Let’s Play”: “Let’s Play is a community partnership led by Canada Dry Mott’s and its parent company. Dr Pepper Snapple Group to get kids and families active…Random Acts of Play is a program that helps facilitate play and healthy, active lifestyles for children all across Canada by donating sports activity equipment to community groups and schools.”	Philanthropy Physical activity Child-targeted
			“ACTION Nation”: “The mission of ACTION Nation is to foster physically active, engaged and sustainable communities where our employees, customers and consumers live and work.”	Philanthropy Physical activity General-targeted
	Cara Operations	3 (11.1)	Alpine Ontario for the K1, K2, and Junior Ski Race Series. “In addition, Swiss Chalet sponsors Alpine Ontario for the K1, K2, and Junior Ski Race Series benefiting almost 4000 kids between the ages of 11 to 19. Partnering with Alpine Ontario gives Swiss Chalet an opportunity to further promote family time both on the hill, as well as in our Restaurants.”	Philanthropic Physical activity Child-targeted
			The Ride to Conquer Cancer “Swiss Chalet is a proud sponsor of The Ride to Conquer Cancer TM benefiting The Princess Margaret Hospital Foundation. The Ride is a unique, two-day cycling event that covers over 200 km from Toronto to Niagara Falls.”	Philanthropic Physical activity General-targeted
			Support to various charities “Kelsey’s, Montana’s, Milestones & Harvey’s partners with charities that support local communities, schools and sports teams across the country.”	Philanthropic Physical activity Child-targeted
	Con-Agra Foods Canada Inc.	1 (3.7)	“Toonies for Tummies”: “Children in your community are going to school hungry - and a toonie can help change that. Snack Pack is proud to sponsor Toonies for Tummies in fighting child hunger and providing nutritious breakfasts to kids across Canada.”	Philanthropy Nutrition Child-targeted
	Dairy Farmers of Canada	7 (25.9)	“Canadian Dairy Research”: “The site contains a vast collection of dairy cattle research results and knowledge at your fingertips. You can access the latest available information on dairy research in formats ranging from scientific articles to news stories. The research results originate from more than 20 years of research funded by Canadian dairy producers in partnership with: Agriculture and Agri-Food Canada, the Natural Sciences and Engineering Research Council, the Canadian Dairy Network and the Canadian Dairy Commission.”	Research Nutrition General-targeted
			“Dairy Nutrition”: “Welcome to Dairy Nutrition, the most comprehensive and up-to-date source of scientific information for the health professional on the role of milk products in nutrition and health.”	Education Nutrition General-targeted
			“Dairy Goodness”: “Make smart choices or to stay in shape. Find out how milk products and better habits can lead you to good health.”	Education Nutrition General-targeted
			“Teach Nutrition”: “Created exclusively to support educators teaching healthy eating.”	Education Nutrition Child-targeted
			Support of School Milk Programs “The Elementary School Milk Program (ESMP) in Ontario, and the School Milk Program (SMP) in New Brunswick and Nova Scotia, help provide students with some of the nutrients they need to stay active and ready to learn all day.”	Philanthropy Nutrition Child-targeted
			The Milk Run “The Milk Run is a 6 km trail run around a dairy farm that will showcase everything amazing about Dairy Farming and Milk!”	Philanthropy Physical activity General-targeted
			Fuelling Women Champions—Champions Fund “The Champions Fund was created by Canada’s dairy farmers to empower the female athletic community, to provide a resource that can help change the game for Canada’s young girls and women athletes.”	Philanthropy Physical activity Child-targeted
	Mccain Foods Canada	1 (3.7)	“McCain Kids Table”: “The McCain Kids Table program works with Breakfast for Learning to help feed hungry children in the community, because we believe no one should go to school hungry.”	Philanthropy Nutrition Child-targeted
	Subway Canada	1 (3.7)	“Expert Advice”: “Nutritionist hired by Subway Canada offering nutritional advice.”	Education Nutrition General-targeted
	Tim Horton’s Canada	2 (7.4)	“Tim Horton’s Free Swim”: “Tim Horton’s restaurant owners proudly offer complimentary swimming during the March Break and summer season at local community pools across the country.”	Philanthropy Physical activity Child-targeted
			The Timbits Minor Sports Program. “The Timbits Minor Sports Program is a community-oriented sponsorship program that provides opportunities for kids aged four to nine to play house league sports.”	Philanthropy Physical activity Child-targeted
	Yum! Brands Inc	1 (3.7)	“Yum! Brands’ World Hunger Relief”: “Pizza Hut has provided over 460 million meals to children around the world through donations raised every year at our restaurants and contributions from our corporate employees.”	Philanthropy Nutrition Child-targeted
	**Total (100)**	**27 (100)**		

The education-oriented CSR initiatives included nutrition information and/or recipes provided on company websites (*n* = 8), participation in a National Nutrition Facts Education Campaign (*n* = 3), the development of educational resources for teachers (*n* = 1) and funding for EatTracker (*n* = 1), a consumer resource developed by Dietitians of Canada that helps track nutrient intake.

A total of 5 initiatives were research-related and mostly involved industry funded non-profits (e.g. The Danone Institute of Canada, the Canadian Foundation for Dietetic Research) or research institutes (e.g. the Gatorade Sports Science Institute) which provide grants and awards for research related to nutrition.

The one initiative classified as “other” involved the reformulation of products to reduce their content in sodium.

### Comparison between the CSR initiatives of CAI and non-CAI companies

Companies participating in the CAI accounted for 57% of identified CSR initiatives (*n* = 36) while non-CAI companies accounted for 43% (*n* = 27). As shown in Table 2, CAI companies had a median number of 1 initiative per company (IQR = 3) while non-CAI companies had a median number of 0 (IQR = 2). This difference was not statistically significant (U = 237; z = 1.434; *p* = .183). CAI companies had a higher proportion of nutrition-related initiatives (72%) and a lower proportion of child-targeted initiatives (42%) compared to non-CAI companies (48 and 54%, respectively) however these differences were not statistically significant (X^2^ = 3.792; df = 1; *p* = .052 and X^2^ = .900; df = 1; *p* = .343, respectively).

Examples of CAI products/brands advertised through CSR activities include, among others, Gatorade (by way of sponsorship of an extreme obstacle course event & Gatorade Hockey camp), Minute Maid 100% fruit juices (through the donation to Breakfast Club of Canada), Nestle Pure Life water (via the sponsorship of a school physical activity program), and Oikos yogurt (through the sponsorship of a yoga event). Examples of non-CAI products/brands promoted through CSR include Swiss Chalet Restaurants (via the sponsorship of children’s skiing competition and a fundraiser for cancer research involving a 2-day cycling event) and Island Farms Milk (by way of donation to school programs), among others.

## Discussion

Among the 39 large food and beverage companies examined, sixty-three CSR initiatives related to nutrition and physical activity were identified on company websites, Facebook pages and annual reports. Most of these initiatives were considered philanthropic or charitable activities which included support to various local, provincial and national organizations and programs, particularly those addressing short-term food insecurity or promoting physical activity among children and youth. Some companies also engaged in nutrition education by providing information on their websites, creating resources for teachers and sponsoring a national consumer education campaign.

Unfortunately, many products sold by the examined food and beverage companies are thought to be incongruent and sometimes in conflict with the health-oriented nature of their CSR initiatives. As such, these initiatives may associate participating companies and their products or brands with positive health attributes, a phenomenon known as the “health halo” effect [15]. Indeed, research has shown that consumers make inferences about the attributes of products based on the perception and reputation of the CSR initiatives with which they are associated [15]. This may impact the consumption choices of those targeted by these initiatives, including children. In fact, CSR initiatives have been shown to be economically beneficial for food and beverage companies and generate increased profits through brand awareness and the health halo effect [26].

### CSR initiatives and children

Overall, our study identified 29 physical activity and nutrition-related CSR initiatives targeting children or youth including, among others, the sponsorship of junior sports teams, events and athletes, financial or food donations to school breakfast programs, and the development of nutrition education materials for school teachers. Though some of these initiatives are less likely to expose children and youth to the products or branding of supporting companies (e.g. financial contribution to children’s charities), many others, such as the sponsorship of children’s sports teams and food donations to school nutrition programs, give food companies the opportunity to directly market to this vulnerable population. In fact, food and beverage marketing, much of which promotes unhealthy products, has been established as a determinant of children’s food preferences and dietary behaviors [27–30]. Research has shown that older youth are also uniquely susceptible to the influence of marketing [31, 32].

Our findings suggest that the practice of promoting physical activity among children and sponsoring their sporting activities is widespread. This is not surprising given that marketing to children and youth through sports has been identified as a particularly powerful marketing technique [33]. Indeed, research on alcohol and tobacco company sponsorship of sporting events has shown that this promotional technique is effective at increasing brand recognition as well as fostering positive attitudes among children and youth towards promoted products or brands [34]. As for food company sponsorship, Kelly et al. (2011) found that most children aged 10–11 years could recall the sponsor of their sports team, held favorable views about this sponsor and said they would like to buy the sponsor’s products to “return the favour” [14]. Similarly, a Canadian study investigating the food experiences of adolescent hockey players revealed that Tim Hortons’ (a large coffee/donut chain) sponsorship of their childhood hockey team had fostered sentiments of loyalty among these youth as well as a desire to support the restaurant in return [35]. In addition to sponsorship, two companies, namely Danone and McDonalds, were also identified as using professional athletes as ambassadors for their initiatives. These types of endorsement strategies are particularly persuasive among children [36]. Unsurprisingly, the use of celebrity athletes has also been shown to have a “health halo” effect whereby influencing the perceived healthfulness of food products, even among adults [37]. By focusing on sports, food companies are shifting the emphasis of healthy living towards physical activity rather than diet. This tactic is common among food and beverage companies as it deflects attention away from their own role in the growing rates of obesity and nutrition-related NCDs and shifts blame onto individuals and their sedentary lifestyle [18, 38, 39].

Consistent with other research [40, 41], we also identified one industry association (Dairy Farmers of Canada) that has developed nutrition education materials for use by teachers, from kindergarten to high school [42]. Though it is not known how widespread their use is, anecdotal evidence suggests that branded educational activities created by food companies are indeed making their way into Canadian classrooms, sometimes unbeknownst to parents [43]. Several companies also reported donating food to school nutrition programs. This may expose children to branded food packaging, an important source of marketing exposure among children [44] and may develop their preferences for donated foods or food categories. This is concerning, particularly if promoted products are unhealthy, as such promotion within schools may create conflicting messages about healthy eating and is antithetical to a school’s mandate to educate and ensure the welfare of students [45]. Children from lower socioeconomic status backgrounds may also be disproportionately impacted by this form of marketing as they likely receive the greatest assistance from these food programs. This may further propagate health inequities among children from lower socioeconomic backgrounds [46, 47]. To our knowledge, no research has examined the nutritional quality of products donated to school nutrition programs. Protecting children from the possible commercial interests of food companies donating food to school nutrition programs provides another argument in support of a government-funded national school food program that many organizations and academics are currently advocating for in Canada [48, 49].

This study also found that companies participating in the CAI were engaged in child-targeted CSR initiatives. For instance, four CAI companies were identified as supporting school nutrition programs, including Coca-Cola and Danone who donate fruit juice and yogurt, respectively, to a charity called Breakfast Club of Canada. Three companies also supported physical activity and sports among children under 12 years, namely Danone who has held a branded international soccer tournament (Danone Nations Cup) for children between the ages of 10 and 12 years, and McDonald’s who sponsors amateur hockey leagues for children aged 10 to 11 years [50, 51]. Though these initiatives do not contravene the pledges made under the CAI, they certainly highlight its inadequacies. Coca-Cola, for example, has pledged to not advertise directly to children in various media and in schools. However, charitable activities are explicitly excluded from the scope of prohibited activities [23]. The company’s donation of fruit juice to school nutrition programs could be considered a form of marketing, much like the distribution of free samples, and may expose children directly to their branded products and promote a food (i.e. beverages with free sugars) that is already overconsumed by Canadian children [52]. The promotion of CAI company brands through sports sponsorship and other health-related CSR initiatives is also problematic. The “health halo” effect created by these initiatives likely extends to all products under the same brand, including those considered unhealthy or uncompliant with the CAI’s own nutrition criteria. As such, these activities undermine the stated purpose of the CAI of promoting healthy choices and lifestyles. The scope of the CAI also excludes the protection of older youth, who are also being targeted by the CSR initiatives of CAI companies including Coca-Cola (e.g. ParticipAction) and PepsiCo (e.g. Gatorade sponsored hockey camp [53].

### Cross-sector engagement and conflicts of interests

Our findings also revealed several instances of cross-sector engagement between food companies and governmental and non-governmental organizations. For instance, three examined companies advertised their participation in the Nutrition Facts Education Campaign on their websites. This national campaign promoted the use and understanding of nutrition labels from 2010 to 2015 and was part of a broader partnership between Food and Consumer Products of Canada, an industry interest group, and Health Canada, the government department responsible for “helping Canadians maintain and improve their health” [54, 55]. This campaign exemplifies the food industry’s tendency to frame obesity as a matter of personal responsibility [18, 38, 39]. Governmental and health organizations should reconsider partnerships of this kind as these may undermine their public image.

Overall, there is much debate regarding the role the food industry should play in the prevention of obesity and NCDs and the appropriateness of cross-sector engagement in this area [56, 57]. Many oppose partnerships between food industry and health organizations because of inherent conflicts of interests [39, 58, 59]. In addition to potentially compromising the credibility of health organizations, partnering with food companies, particularly those that largely produce and promote unhealthy food products, could confer an aura of healthfulness, goodwill and credibility to these industry partners while eclipsing the fact that many of the same companies or their industry associations persistently and aggressively push-back against government policies and the efforts of public health advocates aimed at improving diet and health [60–62]. Some argue that cross-sectoral partnership may also distort the priorities of beneficiary organizations and lead to self-censorship. They may also facilitate industry’s access to regulators or public health leaders whereby increasing opportunities to influence decision-making [56, 57]. As such, the beneficiaries of food industry funding and support should consider the unintended consequences and conflicts of interests that arise from engaging with the food industry and should carefully weigh the risks and benefits associated with such engagement.

Several food companies were also identified as being involved in CSR initiatives that address short-term food insecurity. Their support took on many forms including financial contributions, food donations and participation in a national fundraising campaign called Toonies for Tummies led by the Grocery Foundation [63]. Some argue that such programs reinforce misconceptions about the causes of hunger among the public. Although these programs do meet short term needs, they shift the narrative about the cause of food insecurity towards food shortages rather than emphasize social issues such poverty and powerlessness [64]. They also allow food companies dependant on cheap labor, such as fast food restaurants and grocery chains, to portray themselves as part of the solution while contributing to the issue with their low employee wages [64]. Furthermore, the act of donation itself may not be a wholly selfless practice. Canadian research suggested that food companies or retailers may use food bank donations to dispose of sub-retail standard food products and reduce costs related to waste removal [65].

### Corporate contributions to research funding

Unsurprisingly, some food and beverage companies were also found to provide funding for nutrition and physical activity research. The appropriateness and potential hazards associated with accepting research funding from the food industry is a subject of debate among researchers [66–69]. Legitimate concerns have been raised regarding the risk of conscious and unconscious bias that can result from accepting industry funding [69]. Some evidence has in fact suggested that industry funded research more often lead to conclusions that are in favour of sponsors’ interests. This is particularly true of research examining the link between sugar-sweetened beverages and nutritional health (e.g. [70, 71]). Food and beverage companies have also been accused of using research to emphasize the lack of physical activity as the cause of obesity to shift blame away from their own products [18, 38, 39]. For example, publications funded by Coca-Cola have been shown to emphasize physical activity over sugar intake in relation to weight gain and obesity [18].

Recently, Serôdio, McKee, and Stuckler (2018) also noted a lack in transparency in industry influence on nutrition research literature [18]. In 2015, Coca-Cola published a “Transparency List” of funded research in the USA. However, this list does not seem to comprehensively declare all sponsored activities; 471 authors in 128 studies did not appear in the “Transparency List” but declared funding from Coca-Cola [18]. The reverse was also found to be true with 38 authors listed on Coca-Cola’s “Transparency List” who did not declare any conflicts of interest [18]. This lack of transparency in research funding combined with the biases in funded research has large public health implications. For instance, industry funding could threaten the advancement public health by potentially compromising the reputations of industry-funded researchers and decreasing confidence in their findings due to real or perceived conflicts of interest [69]. It may also divert efforts and resources away from research that is more relevant to public health [72].

### Strengths and limitations

Limitations to the current study must be noted. The companies we examined were not randomly selected; we selected companies committed to the CAI initiative and the top food and beverage advertisers on four selected child and youth television stations. The selection of the latter companies was also made based on media data from 2013 while data on CSR initiatives were collected in 2016. The statistical analyses testing differences between CAI and non-CAI companies should also be interpreted with caution. Each CSR initiatives was counted as one initiative even though they varied widely in nature and scope. In addition, documentation of CSR initiatives was limited to those reported on the company websites, Facebook pages, and corporate annual reports published during a limited time period. This may not represent the true number of initiatives undertaken by these companies as many CSR initiatives can be specific to local communities. Furthermore, the announcement dates of many initiatives identified in this study were not specified. Therefore, it is unknown if all initiatives were being carried out at the time of the study. To get a more comprehensive picture of these initiatives, future research should consider examining additional sources of information such as media articles and industry association websites. Despite these shortcomings, this is one of the few studies to assess food industry CSR initiatives in Canada [73].

## Conclusion

Studies have shown that existing self-regulatory marketing pledges have not significantly improved public health outcomes and that the private sector needs to be held more accountable [74]. Most relevant to the current study, research has shown that self-regulatory initiatives such as the CAI have failed to reduce unhealthy food and beverage advertising to children and legislative restrictions have been proposed as a solution [75–77]. Given that some CSR initiatives expose children to branded products and food company branding, these activities are akin to marketing and should therefore be considered for inclusion in statutory restrictions of unhealthy food marketing to children. The monitoring of CSR initiatives targeted at children is also recommended in order to track any changes in company practices over time.

However, any policies that restrict CSR initiatives of food and beverage companies such as sports sponsorship or donations to food banks would require alternative funding mechanisms; otherwise, these policies may be unpopular and supported programs may not be sustainable [14]. In the past, tobacco and alcohol companies have been prohibited from sponsoring ski events due to it being “not consistent with a healthy family sport like skiing” [78], in a similar fashion, the CSR initiatives of the food industry are not consistent with a healthy lifestyle. A case can be made for encouraging non-food and beverage companies to sponsor health related initiatives and programs. In this way, conflicts between the business interests of sponsoring companies and the public health objectives of sponsored initiatives can be minimized. Advocacy for increased government funding for these programs and for research is crucial as well.

## Supplementary information

**Additional file 1.**

## Data Availability

The datasets used and/or analysed during the current study are available from the corresponding author on reasonable request. The Nielsen data used to identify companies that advertise the most on children’s specialty stations are not publicly available however the data on company initiatives included in this study were collected from websites and documents that were publicly available.

## Abbreviations

CAI: The Canadian Children’s Food and Beverage Advertising Initiative
CSR: Corporate social responsibility
IQR: Interquartile range
NCD: Noncommunicable disease

## References

[1] Abarca-Gómez L, Abdeen ZA, Hamid ZA, Abu-Rmeileh NM, Acosta-Cazares B, Acuin C (2017). Worldwide trends in body-mass index, underweight, overweight, and obesity from 1975 to 2016: a pooled analysis of 2416 population-based measurement studies in 128·9 million children, adolescents, and adults. Lancet..

[2] Pinhas-Hamiel O, Zeitler P (2005). The global spread of type 2 diabetes mellitus in children and adolescents. J Pediatr.

[3] Zhou B, Bentham J, Di Cesare M, Bixby H, Goodarz D, Cowan MJ (2017). Worldwide trends in blood pressure from 1753 to 2015: a pooled analysis of 1479 population-based measurement studies with 19.1 million participants. Lancet.

[4] Janssen I (2013). The public health burden of obesity in Canada. Can J Diabetes.

[5] Lobstein T, Baur L, Uauy R (2004). Obesity in children and young people: a crisis in public health. Obes Rev.

[6] Moodie R, Stuckler D, Monteiro C, Sheron N, Neal B, Thamarangsi T (2013). Profits and pandemics: prevention of harmful effects of tobacco, alcohol, and ultra-processed food and drink industries. Lancet.

[7] Spencer NN (2018). The social determinants of child health. Paediatr Child Health.

[8] Ulijaszek SJ, Pentecost M, Marcus C, Karpe F, Frühbeck G, Nowicka P (2017). Inequality and childhood overweight and obesity: a commentary. Pediatr Obes.

[9] Swinburn BA, Sacks G, Hall KD, McPherson K, Finegood DT, Moodie ML (2011). The global obesity pandemic: shaped by global drivers and local environments. Lancet.

[10] Swinburn BA, Sacks G, Ravussin E (2009). Increased food energy supply is more than sufficient to explain the US epidemic of obesity. Am J Clin Nutr.

[11] Stuckler D, McKee M, Ebrahim S, Basu S (2012). Manufacturing epidemics: the role of global producers in increased consumption of unhealthy commodities including processed foods, alcohol, and tobacco. PLoS Med.

[12] Richards Z, Thomas SL, Randle M, Pettigrew S (2015). Corporate social responsibility programs of big food in Australia: a content analysis of industry documents. Aus N Z J Public Health.

[13] Lee SY, Carroll CE (2011). The emergence, variation, and evolution of corporate social responsibility in the public sphere, 1980-2004: the exposure of firms to public debate. J Bus Ethics.

[14] Kelly B, Baur LA, Bauman AE, Chapman K, Smith BJ (2011). Food company’s,ponsors are kind, generous and cool: (Mis) conceptions of junior sports players. Int J Behavl Nutr Phys Act.

[15] Peloza J, Ye C, Montford WJ (2015). When companies do good, are their products good for you? How corporate social responsibility creates a health halo. J Public Policy Mark.

[16] Richards Z, Phillipson L (2017). Are big Food’s corporate social responsibility strategies valuable to communities? A qualitative study with parents and children. Public Health Nutr.

[17] Powell D (2014). Childhood obesity, corporate philanthropy and the creeping privatisation of health education. Crit Public Health.

[18] Serôdio PM, McKee M, Stuckler D (2018). Coca-Cola - a model of transparency in research partnerships? A network analysis of Coca-Cola’s research funding (2008-2016). Public Health Nutr.

[19] Brownell KD, Warner KE (2009). The perils of ignoring history: big tobacco played dirty and millions died. How similar is big food?. Milbank Q.

[20] Chopra M, Darnton-Hill I (2004). Tobacco and obesity epidemics: not so different after all?. BMJ.

[21] Hawkes C, Lobstein T (2011). Regulating the commercial promotion of food to children: a survey of actions worldwide. Int J Pediatr Obes.

[22] World Cancer Research Fund International. NOURISHING Framework: Restrict food advertising and other foms of commercial promotion. 2019. https://www.wcrf.org/sites/default/files/4_Restrict%20advertising_May2019.pdf Accessed August 6 2019.

[23] Ad Standards Canada. Canadian Children’s Food and Beverage Advertising Initiative. 2018. https://adstandards.ca/wp-content/uploads/2018/11/CCFBAI_EN-Nov-2018.pdf .Accessed August 6 2019.

[24] Kelly B, Baur LA, Bauman AE, King L, Chapman K, Smith BJ (2011). Food and drink sponsorship of children’s sport in Australia: who pays?. Health Promo Int.

[25] Du S, Bhattacharya CB, Sen S (2010). Maximizing business returns to corporate social responsibility (CSR): the role of CSR communication. Int J of Manag Rev.

[26] Ad Standards. The Canadian Children’s Food and Beverage Advertising Initiative. 2016 Compliance Report. https://adstandards.ca/wp-content/uploads/2018/03/2016ComplianceReport-2.pdf. Accessed May 11 2020.

[27] McGinnis JM, Gootman J, Kraak VI. Food marketing to children and youth: threat or opportunity. The National Academies Press: Washington, DC; 2006.

[28] Cairns G, Angus K, Hastings G, Caraher M (2013). Systematic reviews of the evidence on the nature, extent and effects of food marketing to children. A retrospective summary. Appetite.

[29] Sadeghirad B, Duhaney T, Motaghipisheh S, Campbell NR, Johnston BC (2016). Influence of unhealthy food and beverage marketing on children’s dietary intake and preference: a systematic review and meta-analysis of randomized trials. Obes Rev.

[30] Norman J, Kelly B, Boyland E, McMahon AT (2016). The impact of marketing and advertising on food behaviours: evaluating the evidence for a causal relationship. Curr Nutr Rep.

[31] Penchmann C, Levine L, Loughlin S, Frances L (2005). Impulsive and self-conscious: adolescents’ vulnerability to advertising and promotion. J Public Policy Mark.

[32] Leslie FM, Levine LJ, Loughlin SE, Penchmann C. Adolescents psychological & neurobiological development: implications for digital marketing. Centre for digital democracy and Berkeley media studies group: Berkley CA; 2009. http://digitalads.org/sites/default/files/publications/digitalads_leslie_et_al_nplan_bmsg_memo.pdf Accessed August 6 2019.

[33] Bragg MA, Roberto CA, Harris JL, Brownwell KD, Elbel B (2018). Marketing food and beverages to youth through sports. J Adolesc Health.

[34] Kelly B, Baur LA, Bauman AE, King L (2011). Tobacco and alcohol sponsorship of sporting events provide insights about how food and beverage sponsorship may affect children's health. Health Promo J Austr.

[35] Caswell MS, Hanning RM (2018). Adolescent perspectives of the recreational ice hockey food environment and influences on eating behaviour revealed through photovoice. Public Health Nutr.

[36] Smits T, Vandebosch H, Neyens E, Boyland E (2015). The persuasiveness of child-targeted endorsement strategies: a systematic review. Annal Int Commun Assoc.

[37] Dixon H, Scully M, Wakefield M, Kelly B, Donovan R (2011). Parent’s responses to nutrient claims and sports celebrity endorsements on energy-dense and nutrient-poor food: an experimental study. Public Health Nutr.

[38] Barlow P, Serôdio P, Ruskin G, McKee M, Stuckler D (2018). Science organisations and Coca-Cola’s ‘war’ with the public health community: insights from an internal industry document. J Epidemiol Community Health.

[39] Jane B, Gibson K (2017). Corporate sponsorship of physical activity promotion programmes: part of the solution or part of the problem?. J Public Health (Oxf).

[40] Molnar A, Boninger F, Harris MD, Libby K, Fogarty J. Promoting consumption at school: health threats associated with schoolhouse commercialism—the fifteenth annual report on schoolhouse commercializing trends: 2011–2012. National Education Policy Center: Boulder, CO; 2013. http://nepc.colorado.edu/publication/schoolhouse-commercialism-2012. Accessed August 6 2019.

[41] Froese-Germain B, Hawkey C, Larose A. McAdie, P, Shaker P. Commercialism in Canadian schools who’s calling the shots?. Canadian Centre for Policy Alternatives: Ottawa, Canada: 2006. https://www.policyalternatives.ca/sites/default/files/uploads/publications/National_ Office_Pubs/2006/Commercialism_in_Canadian_Schools.pdf. Accessed August 6 2019.

[42] Dairy Farmers of Canada. Teach Nutrition https://dairyfarmersofcanada.ca/en/teachnutrition/ab (no date) Accessed August 6 2019.

[43] Freedhoff Y. Ontario schools inviting Pizza Nova to teach nutrition to kindergarteners is apparently a thing now. Weighty Matters; 2018. http://www.weightymatters.ca/2018/04/ontario-schools-inviting-pizza-nova-to.html Accessed August 6 2019.

[44] Signal LN, Stanley J, Smith M, Barr MB, Chambers TJ, Zhou J (2017). Children’s everyday exposure to food marketing: an objective analysis using wearable cameras. Int J Behav Nutr Phys Act.

[45] Raine G (2007). Commercial activities in primary schools: a quantitative study. Oxford Rev Edu.

[46] Ogden CL, Lamb MM, Carroll MD, Flegal KM (2010). Obesity and socioeconomic status in children and adolescents: United States, 2005-2008. NCHS Data Brief.

[47] Stamatakis E, Wardle J, Cole TJ (2010). Childhood obesity and overweight prevalence trends in England: evidence for growing socioeconomics disparities. Int J Obes.

[48] Coalition for healthy school food Who we are. (no date) https://www.healthyschoolfood.ca/who-we-are. Accessed August 6 2019.

[49] Hernandez K, Engel-Stringer R, Kirk S, Wittman H, McNicholl S (2018). The case for a Canadian national school food program. Can Food Stud.

[50] Danone Nation Cup. Available from: https://www.danonenationscup.com/en/. Accessed April 2 2020.

[51] McDonalds. AtoMc Hockey. Available from: https://www.mcdonalds.com/ca/en-ca/community/atomchockey.html. Accessed April 2 2020.

[52] Garriguet D (2008). Beverage consumption of children and teens. Health Rep.

[53] Gatorade Canada. Available from: https://www.facebook.com/GatoradeCanada/videos/10154308447263774/ Accessed April 2 2020.

[54] Health Canada. Government of Canada Launches New Phase of Nutrition Facts Education Campaign. Health Canada: Toronto, ON; 2015. https://www.canada.ca/en/news/archive/2015/05/government-canada-launches-new-phase-nutrition-facts-education-campaign.html Accessed August 6 2019.

[55] Food and Consumer Products Canada. Nutrition Facts Education Campaign. (no date) https://www.fcpc.ca/Priorities-Policy/Health-Wellness/Nutrition-Facts-Education-Campaign Accessed August 6 2019.

[56] Hernandez-Aguado I, Zaragoza GA. Support of public-private partnerships in health promotion and conflicts of interest. BMJ Open. 2016:e009342. 10.1136/bmjopen-2015-009342 PMC4838703 27091816

[57] Hawkes C, Buse K. Public-private engagement for diet and health: Addressing the governance gap. SCN News 2010; 39: 6–10. http://www.unscn.org/files/Publications/SCN_News/SCNNEWS39_10.01_high_def.pdf Accessed August 6 2019.

[58] Herrick C (2009). Shifting blame/selling health: corporate social responsibility in the age of obesity. Soc Health Illn.

[59] Johnston LM, Finegood DT (2015). Cross-sector partnerships and public health: challenges and opportunities for addressing obesity and noncommunicable diseases through engagement with the private sector. Annu Rev Public Health.

[60] Pomeranz JL, Zellers L, Bare M, Pertschuk JD (2019). Sate preemption of food and nutrition policies and litigation: undermining government’s role in public health. Am J Prev Med.

[61] Wiist W. The corporate playbook, health, and democracy: the snack food and beverage industry’s tactics in context. In: Stuckler D, Siegel K, editors*.* Sick societies. Oxford, UK: Oxford University Press; 2011. p. 204–216.

[62] Cullerton K, Donnet T, Lee A, Gallegos D (2016). Playing the policy game: a review of the barriers to and enablers of nutrition policy change. Public Health Nutr.

[63] The Grocery Foundation. Toonies for Tummies. 2018. http://www.groceryfoundation.com/tooniesfortummies. Accessed August 6 2019.

[64] Fisher A, Jayaraman S. Big Hunger. Cambridge, USA: The MIT Press; 2017.

[65] Tarasuk V, Eakin JM (2005). Food assistance through “surplus” food: insights from an ethnographic study of food bank work. Agric Human Values.

[66] Poli A, Marangoni F, Agostoni CV, Brancati F, Capurso L, Colombo ML, Ghiselli A, La Vecchia C, Molinari E, Morelli L, Porrini M, Visioli F, Riccardi G (2018). Research interactions between academia and food companies: how to improve transparency and credibility of an invevitable liaison. Eur J Nutr.

[67] Nestle M (2016). Food industry funding of nutrition research. The relevance of history for current debates. JAMA Intern Med.

[68] Aveyard P, Yach D, Gilmore AB, Capewell S (2016). Should we welcome food industry funding of health research?. BMJ.

[69] Nestle M. Unsavory truth. How food companies skew the science of what we eat. New York, USA: Basic Book; 2018.

[70] Litman EA, Gortmaker SL, Ebbeling CB, Lundwig DS (2018). Source of bias in sugar-sweetened beverage research: a systematic review. Public Health Nutr.

[71] Bes-Rastrollo M, Schulze MB, Ruiz-Canela M, Martinez-Gonzalez MA (2013). Financial conflicts of interests and reporting bias regarding the association between sugar-sweetened beverages and weight gain: a systematic review of systematic reviews. PLoS Med.

[72] Fabbri A, Holland TJ, Bero LA (2018). Food industry sponsorship of academic research: investigating commercial bias in the research agenda. Public Health Nutr.

[73] Vanderlee L, Vergeer L, Sacks G, Robinson E, L’Abbé M. Food and beverage manufacturer in Canada: Policies and commitments to improve the food environment. University of Toronto: Toronto, Canada; 2019. Available from: http://labbelab.utoronto.ca/wp-content/uploads/2019/03/2019-BIA-Obesity-Canada-Food-and-Beverage-Manufacturers.pdf Accessed August 6 2019.

[74] Sacks G, Swinburn B, Kraak V, Downs D, Walker C, Barquera S (2013). A proposed approach to monitor private-sector policies and practices related to food environments, obesity and non-communicable disease prevention. Obes Rev.

[75] Potvin Kent M, Wanless A (2014). The influence of the Children’s food and beverage advertising initiative: change in children’s exposure to food advertising on television in Canada between 2006-2009. Int J Obes.

[76] Potvin Kent M, Smith JR, Pauzé E, L’Abbé M (2018). The effectiveness of the food and beverage industry’s self-established uniform nutrition criteria at improving the healthfulness of food advertising viewed Canadian children on television. Int J Behav Nutr Phys Act.

[77] Potvin Kent M, Pauzé E (2018). The effectiveness of self-regulation in limiting the advertising of unhealthy foods and beverages on children’s preferred websites in Canada. Public Health Nutr.

[78] Crompton JL. Sponsorship of sport by tobacco and alcohol companies: a review of the issues. J Sport Soc Issues. 1993:17148–67.

