# Supplementary material for: The physical activity and nutrition-related corporate social responsibility initiatives of food and beverage companies in Canada and implications for public health
Source: BMC Public Health. 2020 Jun 9;20:890. doi: 10.1186/s12889-020-09030-8 (PMC7281932; doi:10.1186/s12889-020-09030-8)
Supplement: Supplementary file 1 — Additional file 1. [file 12889_2020_9030_MOESM1_ESM.docx]

**Title:** The physical activity and nutrition-related corporate social responsibility initiatives of food and beverage companies in Canada and implications for public health

**Authors:** M. Potvin Kent, E. Pauzé, K. Guo, A. Kent, J. Jean-Louis

**Supplemental Table 1**

| **CAI/**  **Non-CAI** | **Company** | **Source** | **Link** |
| --- | --- | --- | --- |
| CAI | Campbell Company of Canada | Website | <http://www.campbellsoup.ca/en-ca/> |
|  |  | Facebook | <https://www.facebook.com/CampbellCanada/> |
|  |  | Facebook | https://www.facebook.com/GoldfishCA/ |
| CAI | Coca-Cola Canada Ltd. | Website | [http://coca-cola.ca/index.html](http://www.campbellsoup.ca/en-ca/) |
|  |  | Facebook | <https://www.facebook.com/cocacolacanada/> |
| CAI | Danone Canada Inc. | Website | [https://www.danone.ca](https://www.danone.ca/) |
|  |  | Website | <http://www.activia.ca/en> |
|  |  | Website | https://www.dan-on.com/ca-en/first-visit |
|  |  | Facebook | https://www.facebook.com/danonecanada/ |
|  |  | Facebook | https://www.facebook.com/activiaca/ |
|  |  | Facebook | https://www.facebook.com/OikosCanada/ |
|  |  | Facebook | https://www.facebook.com/DanetteCA/ |
| CAI | Ferrero Canada Ltd. | Website | [http://www.ferrero.ca](http://www.ferrero.ca/) |
|  |  | Website | http://www.nutella.com/en/ca/ |
|  |  | Website | https://www.tictaccanada.com/en/ |
|  |  | Website | <http://ferrerorocher.ca> |
|  |  | Facebook | https://www.facebook.com/nutellacanada/ |
|  |  | Facebook | https://www.facebook.com/tictaccanada/?brand_redir=138803372817598 |
|  |  | Facebook | https://www.facebook.com/FerreroRocherCanada |
|  |  | Facebook | https://www.facebook.com/KinderCanada |
| CAI | General Mills Canada Corporation | Website | [http://www.generalmills.ca](http://www.generalmills.ca/) |
|  |  | Website | http://www.naturevalley.ca |
|  |  | Website | http://sendyourcheer.ca |
|  |  | Facebook | <https://www.facebook.com/NesquikCanada/> |
|  |  | Facebook | <https://www.facebook.com/LifeMadeDelicious> |
| CAI | Hershey Canada Inc. | Website | <https://www.hersheycanada.com/en/home> |
|  |  | Website | http://www.brooksidechocolate.com/canadaen/ |
|  |  | Facebook | https://www.facebook.com/hersheyscanada/ |
|  |  | Facebook | https://www.facebook.com/OhHenryCanada/ |
|  |  | Facebook | https://www.facebook.com/reesecanada/ |
|  |  | Facebook | <https://www.facebook.com/Brookside-Chocolate-865032446912091/?brand_redir=118525178203216> |
| CAI | Kellogg's Canada Inc. | Website | <http://www.kelloggs.ca/en_CA/home.html> |
|  |  | Website | https://www.clubkelloggs.ca/en/ |
|  |  | Website | https://www.allbran.ca/en_CA/home |
|  |  | Website | http://www.ricekrispies.ca/en_CA/home.html |
|  |  | Website | http://www.specialk.ca/en_ca/home-page.html |
|  |  | Website | http://www.kelloggsvector.ca/en_CA/home.html |
|  |  | Website | http://www.getyourmoveon.com/?# |
|  |  | Facebook | https://www.facebook.com/AllBranCanada/ |
|  |  | Facebook | https://www.facebook.com/SpecialKCanada/?fref=nf |
|  |  | Facebook | https://www.facebook.com/MiniWheatsCanada/ |
|  |  | Facebook | https://www.facebook.com/kelloggskrave/ |
|  |  | Facebook | https://www.facebook.com/whatscooking/ |
| CAI | Kraft Canada Inc. | Website | [http://www.kraftcanada.ca](http://www.kraftcanada.ca/) |
|  |  | Website | http://www.nabob.ca/en |
|  |  | Website | https://www.tassimo.ca/en/ |
|  |  | Website | http://unclebens.ca |
|  |  | Facebook | https://www.facebook.com/NabobCoffeeCo/ |
|  |  | Facebook | https://www.facebook.com/TASSIMOCanada/ |
|  |  | Facebook | https://www.facebook.com/PhillyCanada/ |
|  |  | Facebook | https://www.facebook.com/KraftPeanutButter/ |
|  |  | Facebook | <https://www.facebook.com/unclebenscanada/> |
| CAI | Maple Leaf Foods | Website | [http://www.mapleleaffoods.com](http://www.mapleleaffoods.com/) |
|  |  | Website | [http://www.larsen.ca](http://www.larsen.ca/) |
|  |  | Website | [http://www.minahalal.com](http://www.minahalal.com/) |
|  |  | Website | [http://www.mapleleafprime.ca](http://www.mapleleafprime.ca/) |
|  |  | Website | [http://www.schneiders.ca](http://www.schneiders.ca/) |
|  |  | Website | [http://www.greenfieldmeat.ca](http://www.greenfieldmeat.ca/) |
|  |  | Facebook | <https://www.facebook.com/MapleLeafFoods/> |
|  |  | Facebook | <https://www.facebook.com/LarsenFoods/> |
|  |  | Facebook | <https://www.facebook.com/MinaHalal/> |
|  |  | Facebook | <https://www.facebook.com/Schneiders/> |
|  |  | Facebook | https://www.facebook.com/Hygrade |
|  |  | Facebook | <https://www.facebook.com/Greenfieldfoods> |
|  |  | Report | <https://www.mapleleaffoods.com/wp-content/uploads/2017/02/Annual-report-for-SEDAR.pdf> |
| CAI | Mars Canada | Website | <http://www.mars.com/canada/en/> |
|  |  | Facebook | <https://www.facebook.com/SnickersCanada/> |
|  |  | Facebook | <https://www.facebook.com/CadburyDairyMilkCanada> |
| CAI | McDonald's Restaurants of Canada Limited | Website | <http://www.mcdonalds.ca/ca/en.html> |
|  |  | Facebook | <https://www.facebook.com/McDonaldsCanada/> |
| CAI | Mondelez Canada | Website | [http://www.mondelezinternational.com](http://www.mondelezinternational.com/) |
|  |  | Website | [http://oreowonderfilled.ca](http://oreowonderfilled.ca/) |
|  |  | Facebook | <https://www.facebook.com/HallsCanada/> |
|  |  | Facebook | [https://www.facebook.com/RitzCanada/#](https://www.facebook.com/RitzCanada/) |
| CAI | Nestle | Website | <https://www.corporate.nestle.ca/en> |
|  |  | Website | <http://www.nestea.ca/> |
|  |  | Website | <https://www.kitkat.ca/en/> |
|  |  | Website | [https://shop.nescafe.ca](https://shop.nescafe.ca/) |
|  |  | Website | [https://www.dolce-gusto.ca](https://www.dolce-gusto.ca/) |
|  |  | Website | <https://www.leancuisine.ca/en/> |
|  |  | Website | <http://www.nestle-waters.ca/en> |
|  |  | Website | <https://www.haagen-dazs.ca/hd-en> |
|  |  | Facebook | <https://www.facebook.com/nestle.ca/?brand_redir=24287259392> |
|  |  | Facebook | <https://www.facebook.com/CBECanada/> |
|  |  | Facebook | <https://www.facebook.com/NesfrutaCanada/> |
|  |  | Facebook | <https://www.facebook.com/aerocanada/> |
|  |  | Facebook | <https://www.facebook.com/NESTEA.CA/> |
|  |  | Facebook | <https://www.facebook.com/NesquikCanada/> |
|  |  | Facebook | <https://www.facebook.com/KITKATCanada/> |
|  |  | Facebook | <https://www.facebook.com/NestleTurtles/> |
|  |  | Facebook | <https://www.facebook.com/Nescafe.CA/> |
|  |  | Facebook | <https://www.facebook.com/LeanCuisineCanada/> |
|  |  | Facebook | <https://www.facebook.com/NestlePureLifeCanada/> |
|  |  | Facebook | <https://www.facebook.com/SkinnyCowCanada/> |
|  |  | Facebook | <https://www.facebook.com/drumstick/> |
|  |  | Facebook | <https://www.facebook.com/haagendazscanada> |
| CAI | Parmalat Canada | Website | [http://corporate.parmalat.ca](http://corporate.parmalat.ca/) |
|  |  | Website | [http://www.lactantia.ca](http://www.lactantia.ca/) |
|  |  | Website | http://www.astrokik.ca |
|  |  | Website | [http://beatrice.ca](http://beatrice.ca/) |
|  |  | Website | [http://www.sensationalsoy.ca](http://www.sensationalsoy.ca/) |
|  |  | Website | [http://www.blackdiamond.ca](http://www.blackdiamond.ca/) |
|  |  | Website | [http://www.cheestrings.ca](http://www.cheestrings.ca/) |
|  |  | Website | [http://baldersoncheese.ca](http://baldersoncheese.ca/) |
|  |  | Website | [http://www.galbani.ca](http://www.galbani.ca/) |
|  |  | Website | [http://www.astro.ca](http://www.astro.ca/) |
|  |  | Facebook | <https://www.facebook.com/lactantiadairy/> |
|  |  | Facebook | <https://www.facebook.com/astrokik/> |
|  |  | Facebook | <https://www.facebook.com/blackdiamondcheese/> |
|  |  | Facebook | <https://www.facebook.com/cheestringsficello/> |
|  |  | Facebook | https://www.facebook.com/Balderson-Cheese-261758450627946/ |
|  |  | Facebook | <https://www.facebook.com/GalbaniCanada> |
|  |  | Facebook | <https://www.facebook.com/astroyogourt/> |
| CAI | Pepsico Canada | Website | <http://pepsico.ca/en/Index.html> |
|  |  | Website | [https://pepsi.ca](https://pepsi.ca/) |
|  |  | Website | [https://pepsinext.ca](https://pepsinext.ca/) |
|  |  | Website | [http://www.aquafina.ca](http://www.aquafina.ca/) |
|  |  | Website | [http://www.lays.ca](http://www.lays.ca/) |
|  |  | Website | [http://www.spitz.ca](http://www.spitz.ca/) |
|  |  | Website | [http://www.stacyssnacks.ca](http://www.stacyssnacks.ca/) |
|  |  | Website | [http://www.sunchips.ca](http://www.sunchips.ca/) |
|  |  | Website | [http://www.tostitos.ca](http://www.tostitos.ca/) |
|  |  | Website | [http://www.twistos.ca](http://www.twistos.ca/) |
|  |  | Website | [https://www.tropicana.ca](https://www.tropicana.ca/) |
|  |  | Website | [http://www.crispyminis.ca](http://www.crispyminis.ca/) |
|  |  | Website | [http://www.quakeroats.ca](http://www.quakeroats.ca/) |
|  |  | Website | [http://www.gatorade.ca](http://www.gatorade.ca/) |
|  |  | Website | [http://dolecanada.ca](http://dolecanada.ca/) |
|  |  | Website | [http://www.oceanspray.ca](http://www.oceanspray.ca/) |
|  |  | Website | [http://www.liptontea.ca](http://www.liptontea.ca/) |
|  |  | Facebook | <https://www.facebook.com/PepsiCanada> |
|  |  | Facebook | <https://www.facebook.com/BriskCanada/> |
|  |  | Facebook | <https://www.facebook.com/MountainDewCanada/> |
|  |  | Facebook | <https://www.facebook.com/DoritosCanada/> |
|  |  | Facebook | <https://www.facebook.com/LaysCanada/> |
|  |  | Facebook | <https://www.facebook.com/RufflesCanada/> |
|  |  | Facebook | <https://www.facebook.com/smartfoodcanada/> |
|  |  | Facebook | <https://www.facebook.com/tostitoscanada/> |
|  |  | Facebook | <https://www.facebook.com/TwistosCanada/> |
|  |  | Facebook | <https://www.facebook.com/TropicanaCanada/> |
|  |  | Facebook | <https://www.facebook.com/quakercanada> |
|  |  | Facebook | <https://www.facebook.com/GatoradeCanada/> |
|  |  | Facebook | https://www.facebook.com/OceanSprayCanada/?brand_redir=117917151585263 |
|  |  | Facebook | <https://www.facebook.com/LiptonCanada> |
|  |  | Facebook | <https://www.facebook.com/DoleCanada> |
| CAI | Post Foods Canada Corporation | Website | [http://www.postfoods.ca](http://www.postfoods.ca/) |
|  |  | Facebook | <https://www.facebook.com/PostFoodsCanada/> |
| CAI | Unilever Food Division | Website | [https://www.unilever.ca](https://www.unilever.ca/) |
|  |  | Website | <http://www.becel.ca/en/> |
|  |  | Website | [http://www.hellmanns.ca](http://www.hellmanns.ca/) |
|  |  | Website | [http://www.knorr.ca](http://www.knorr.ca/) |
|  |  | Website | <http://www.benandjerrys.ca/en/> |
|  |  | Facebook | <https://www.facebook.com/Becel/> |
|  |  | Facebook | <https://www.facebook.com/HellmannsCanada> |
|  |  | Facebook | <https://www.facebook.com/KnorrCanada/> |
|  |  | Facebook | <https://www.facebook.com/LiptonCanada> |
|  |  | Facebook | <https://www.facebook.com/BenJerryCanada/> |
| CAI | Weston Foods Canada | Website | [http://www.westonfoods.ca](http://www.westonfoods.ca/) |
|  |  | Website | [http://www.wonderbread.ca](http://www.wonderbread.ca/) |
|  |  | Website | <http://www.ditaliano.ca/products/1> |
|  |  | Website | <http://www.gadoua.qc.ca/fr/> |
|  |  | Website | <http://countryharvest.com/en/> |
|  |  | Website | [http://allbutgluten.ca](http://allbutgluten.ca/) |
|  |  | Website | <http://fourneeweston.ca/en> |
|  |  | Website | [http://www.acebakery.ca](http://www.acebakery.ca/) |
|  |  | Website | [http://www.flatovenbakery.com](http://www.flatovenbakery.com/) |
|  |  | Facebook | <https://www.facebook.com/Wonderbreadcanada/> |
|  |  | Facebook | <https://www.facebook.com/ditaliano/> |
|  |  | Facebook | <https://www.facebook.com/countryharvestbread/timeline> |
|  |  | Facebook | <https://www.facebook.com/GlutenFreeAllButGluten/> |
|  |  | Facebook | <https://www.facebook.com/ACEBakery/> |
|  |  | Report | <http://www.weston.ca/en/pdf_en/gwl_2016ar_en.pdf> |
| Non-CAI | Agropur Co-Op | Website | <http://www.agropur.com/en/> |
|  |  | Website | <http://www.iogo.ca/en/> |
|  |  | Website | [http://www.ultimayog.ca](http://www.ultimayog.ca/) |
|  |  | Website | <https://www.olympicdairy.com/home> |
|  |  | Website | <http://ancocheeses.ca/intro> |
|  |  | Website | <http://www.centraldairies.com/pages/index.php> |
|  |  | Website | <http://www.dairytown.com/> |
|  |  | Website | [http://www.northumberlanddairy.ca](http://www.northumberlanddairy.ca/) |
|  |  | Website | <http://www.quebon.ca/en/> |
|  |  | Website | [http://www.sealtest.ca](http://www.sealtest.ca/) |
|  |  | Website | <http://www.pleasureandcheeses.ca/> |
|  |  | Website | <http://www.natrel.ca/en> |
|  |  | Website | [http://islandfarms.com](http://islandfarms.com/) |
|  |  | Website | [http://www.myallegro.ca](http://www.myallegro.ca/) |
|  |  | Website | <http://www.grandcheddar.ca/en/> |
|  |  | Facebook | <https://www.facebook.com/iogo/> |
|  |  | Facebook | <https://www.facebook.com/OlympicDairy/> |
|  |  | Facebook | <https://www.facebook.com/NorthumberlandDairy/> |
|  |  | Facebook | <https://www.facebook.com/natrel/> |
|  |  | Facebook | <https://www.facebook.com/islandfarmsdairy/> |
|  |  | Facebook | <https://www.facebook.com/FarmersDairy> |
|  |  | Report | <https://www.agropur.com/sites/default/files/2020-03/2016_anglais_modifi%C3%A9.pdf> |
| Non-CAI | Arla Foods Inc. | Website | [http://www.arlafoods.ca](http://www.arlafoods.ca/) |
|  |  | Website | <https://www.trestelle.ca/english/home> |
|  |  | Website | <http://www.castellocheese.com/en-ca> |
|  |  | Facebook | <https://www.facebook.com/ArlaCanada/?fref=ts> |
|  |  | Facebook | <https://www.facebook.com/TreStelle/> |
|  |  | Facebook | <https://www.facebook.com/CastelloCanada> |
| Non-CAI | A&W Food Services of Canada Inc. | Website | [http://www.aw.ca](http://www.aw.ca/) |
|  |  | Facebook | <https://www.facebook.com/AWCanada/> |
| Non-CAI | Burger King Corp. | Website | [http://burgerking.ca](http://burgerking.ca/) |
|  |  | Facebook | <https://www.facebook.com/BurgerKingCanada/> |
| Non-CAI | Canada Dry Mott’s Inc. (Dr. Pepper Snapple Group) | Website | [http://www.canadadrymotts.ca](http://www.canadadrymotts.ca/) |
|  |  | Website | <http://www.drpepper.ca/en/> |
|  |  | Website | [http://www.mottsclamato.ca](http://www.mottsclamato.ca/) |
|  |  | Website | [http://www.mottsfruitsations.ca](http://www.mottsfruitsations.ca/) |
|  |  | Website | [http://www.schweppes.ca](http://www.schweppes.ca/) |
|  |  | Website | [http://www.crushcanada.ca](http://www.crushcanada.ca/) |
|  |  | Website | [http://www.canadadry.ca](http://www.canadadry.ca/) |
|  |  | Facebook | <https://www.facebook.com/drpeppercanada/> |
|  |  | Facebook | <https://www.facebook.com/mottsclamatocaesar> |
|  |  | Facebook | <https://www.facebook.com/FruitsationsCanada/> |
|  |  | Facebook | <https://www.facebook.com/CrushCanada> |
|  |  | Facebook | <https://www.facebook.com/CanadaDryCanada> |
| Non-CAI | Cara Operations LTD. | Website | [https://www.cara.com](https://www.cara.com/) |
|  |  | Website | <http://www.eastsidemarios.com/> |
|  |  | Website | <http://www.caseysgrillbar.com/> |
|  |  | Website | <http://www.primepubs.com/> |
|  |  | Website | <http://www.thebiermarkt.com/> |
|  |  | Website | <http://www.harveys.ca/> |
|  |  | Website | <http://www.kelseys.ca/> |
|  |  | Website | <http://www.montanas.ca/> |
|  |  | Website | <http://www.milestonesrestaurants.com/home.php> |
|  |  | Website | <https://www.swisschalet.com/> |
|  |  | Website | <http://www.newyorkfries.com/locations/all> |
|  |  | Facebook | <https://www.facebook.com/eastsidemarios/> |
|  |  | Facebook | <https://www.facebook.com/CaseysGrillBar/> |
|  |  | Facebook | <https://www.facebook.com/BierMarkt/> |
|  |  | Facebook | <https://www.facebook.com/HarveysCanada/> |
|  |  | Facebook | <https://www.facebook.com/KelseysRestaurants/> |
|  |  | Facebook | <https://www.facebook.com/Montanasribs/> |
|  |  | Facebook | <https://www.facebook.com/milestonesrestaurants/> |
|  |  | Facebook | <https://www.facebook.com/SwissChalet/> |
|  |  | Facebook | <https://www.facebook.com/NewYorkFries/> |
|  |  | Report | <https://recipeunlimited.investorroom.com/annual-reports> |
| Non-CAI | Con-Agra Foods Canada Inc. | Website | [http://www.conagrafoods.ca](http://www.conagrafoods.ca/) |
|  |  | Website | <http://www.aylmeraccents.ca/> |
|  |  | Website | <http://www.chefboyardee.ca/en/> |
|  |  | Website | <https://healthychoicecanada.conagrafoods.ca/> |
|  |  | Website | <http://huntstomatoes.conagrafoods.ca/> |
|  |  | Website | <http://orville.conagrafoods.ca/> |
|  |  | Website | <http://www.snackpackcanada.ca/> |
|  |  | Website | <http://vhsauces.conagrafoods.ca/> |
|  |  | Website | <http://vhsauces.conagrafoods.ca/en/steamers.html> |
|  |  | Facebook | <https://www.facebook.com/chefboyardeecanada/> |
|  |  | Facebook | <https://www.facebook.com/HealthyChoiceCanada/> |
|  |  | Facebook | <https://www.facebook.com/OrvilleCanada/> |
|  |  | Facebook | <https://www.facebook.com/snackpackcanada/> |
|  |  | Facebook | <https://www.facebook.com/VHSteamers/> |
| Non-CAI | Dairy Farmers of Canada | Website | <https://www.dairyfarmers.ca/> |
|  |  | Website | [https://www.womenchampions.ca](https://www.womenchampions.ca/) |
|  |  | Website | [https://www.dairynutrition.ca](https://www.dairynutrition.ca/) |
|  |  | Website | [https://www.dairyresearch.ca](https://www.dairyresearch.ca/) |
|  |  | Website | <https://www.dairygoodness.ca/good-health> |
|  |  | Website | [http://www.teachnutrition.ca](http://www.teachnutrition.ca/) |
|  |  | Facebook | <https://www.facebook.com/dfcplc/> |
|  |  | Report | <https://dairyfarmersofcanada.ca/sites/default/files/2019-07/corp%20DFC%20Annual%20Report%202016%202017_0_0.pdf> |
| Non-CAI | Dairy Queen Restaurant | Website | http://www.dairyqueen.com/ca-en/ |
|  |  | Facebook | https://www.facebook.com/dairyqueencanada/?brand_redir=15508591668 |
| Non-CAI | Dare Foods | Website | <http://www.darefoods.com/ca_en> |
|  |  | Facebook | <https://www.facebook.com/BearPaws.PattesDours> |
|  |  | Facebook | <https://www.facebook.com/Breton> |
|  |  | Facebook | <https://www.facebook.com/BoulangerieGrissol> |
|  |  | Facebook | <https://www.facebook.com/DareCandyCo?fref=ts> |
|  |  | Facebook | <https://www.facebook.com/monwhippet/> |
| Non-CAI | Darden Restaurants Inc. | Website | <http://www.olivegarden.ca/home> |
| Non-CAI | Fromageries Bel Sa | Website | <http://www.groupe-bel.com/en/> |
|  |  | Website | <http://www.boursin.ca/en/> |
|  |  | Website | <http://www.minibabybel.ca/en-ca> |
|  |  | Website | [http://www.lavachequirit.ca](http://www.lavachequirit.ca/) |
|  |  | Facebook | <https://www.facebook.com/BoursinCanada>/ |
| Non-CAI | Hormel Foods | Website | [http://www.hormel.ca](http://www.hormel.ca/) |
|  |  | Facebook | <https://www.facebook.com/StaggChiliCanada> |
| Non-CAI | McCain Foods Canada | Website | <http://mccain.ca/en> |
|  |  | Facebook | <https://www.facebook.com/mccaincanada?fref=ts> |
| Non-CAI | Quizno's Corp. | Website | <http://www.quiznos.ca/Home.aspx> |
|  |  | Facebook | <https://www.facebook.com/QuiznosCanada> |
| Non-CAI | Redbull LTD. | Website | <http://energydrink-ca.redbull.com/en> |
| Non-CAI | Storck International | Website | <https://www.storck.ca/en/> |
| Non-CAI | Subway Canada | Website | <http://w.subway.com/en-ca/> |
|  |  | Facebook | <https://www.facebook.com/SubwayCanada/> |
| Non-CAI | Tim Horton's Canada | Website | <http://www.timhortons.com/ca/en/index.php> |
|  |  | Facebook | <https://www.facebook.com/TimHortons/> |
| Non-CAI | Wendy's Company | Website | <https://www.wendys.com/en-ca> |
|  |  | Facebook | <https://www.facebook.com/WendysCanada> |
| Non-CAI | Yum! Brands Inc/ KFC | Website | [http://www.yum.com](http://www.yum.com/) |
|  |  | Website | https://www.pizzahut.ca/ |
|  |  | Website | [http://www.kfc.ca](http://www.kfc.ca/) |
|  |  | Website | <http://www.tacobell.ca/en> |
|  |  | Facebook | <https://www.facebook.com/pizzahutcanada/> |
|  |  | Facebook | <https://www.facebook.com/KFCCanada> |
